# Cinnamaldehyde Alleviates Salmonellosis in Chicks by Regulating Gut Health

**DOI:** 10.3390/vetsci12030237

**Published:** 2025-03-03

**Authors:** Lizi Yin, Luxin Li, Xue Lv, Fengsheng Sun, Yuyun Dai, Yingzi Guo, Shihao Peng, Chenyu Ye, Xiaoxia Liang, Changliang He, Gang Shu, Ping Ouyang

**Affiliations:** Department of Basic Veterinary Medicine, Sichuan Agricultural University, Chengdu 611100, China; yinlizi@sicau.edu.cn (L.Y.); luckypupu99@163.com (L.L.); lvxue9647@163.com (X.L.); sss44660@126.com (F.S.); dyy15928065710@163.com (Y.D.); 18154560276@163.com (Y.G.); psh880889@163.com (S.P.); 18857813616@163.com (C.Y.); liangxiaoxia@sicau.edu.cn (X.L.); lorri190@126.com (C.H.); cndyx2005@163.com (G.S.)

**Keywords:** cinnamaldehyde, pullorum disease, intestinal tight junction, gut microbial, metabolomics

## Abstract

Due to the high mortality rate in chicks caused by pullorum disease (PD) and the drawbacks of antibiotic resistance, the poultry industry is increasingly interested in using natural herbal antimicrobial agents as alternatives, with cinnamaldehyde (CA) being a focus due to its multitarget and synergistic effects. This study aimed to evaluate the effects of oral administration of CA on restoring intestinal physical integrity, intestinal microbial barrier, and intestinal metabolism in a laboratory model of *Salmonella pullorum* (*S. pullorum*) infection in chicks. Thirty-six chicks were divided into six groups. The S.P and CA groups were infected with 5 × 10^8^ CFU/mL, 0.5 mL *S. pullorum*, while the CON group received an equal-volume saline injection. The CA group was treated with 100 mg/kg CA, and the others received phosphate buffer saline (PBS). Samples were collected 24 h after the last treatment. Intestinal physical integrity was assessed by H&E staining, and ELISA was used to measure inflammatory factors. In situ hybridization (ISH) and RT-qPCR were used to measure the expression of tight-junction protein mRNA. The microbiota was analyzed by 16S rRNA gene sequencing of the ileal contents, and metabolite analysis was performed on the intestinal contents. After CA treatment, the expression of IL-1β and TNF-α was reduced, and IL-10 was increased (*p* < 0.05). H&E staining showed that the intestinal structure was partially restored after treatment. ISH results showed that the fluorescence intensity indicating gene expression status was low in the S.P group and high in the CA group, indicating reduced intestinal permeability. RT-qPCR showed that CA up-regulated the mRNA expression of tight-junction proteins (claudin-1, occludin-1, and zo-1, *p* < 0.05). The 16S rRNA gene sequence analysis showed that *Salmonella* was significantly enriched in the S.P group (LDA score > 2.0, *p* < 0.05), while specific genera were significantly more abundant in the treated groups. Untargeted sequencing of intestinal contents showed that key metabolites (butyrate, alanine, glutamate, cholesterol, and propionate) in the CA group were significantly changed compared with the S.P group (*p* < 0.05). CA treatment was the most effective method for reducing PD intestinal colonization and maintaining better intestinal homeostasis, possibly by regulating intestinal microbiota and metabolic functions.

## Simple Summary

This study investigated the efficacy of cinnamaldehyde (CA) in the treatment of pullorum disease caused by *Salmonella pullorum* (*S. pullorum*) infection. Specifically, CA prevents further spread of *S. pullorum* by repairing damaged intestinal tight junctions. Additionally, CA ameliorated the disorganization of gut microbial structure caused by *S. pullorum* infection, remodels intestinal metabolism, and thereby restores chick health. In conclusion, our results suggest that CA may serve as an effective treatment for pullorum disease. In addition, this manuscript provides valuable information for further research.

## 1. Introduction

Pullorum disease (PD), caused by *Salmonella pullorum* (*S. pullorum*), is an infectious disease that primarily affects chicks and is recognized as one of the most damaging diseases to the chicken industry due to its high mortality rates in chicks [1,2]. Currently, pharmacological interventions, including antibiotics such as sulfonamides and aminoglycosides, are commonly employed to inhibit *Salmonella* proliferation and alleviate clinical symptoms [3,4]. However, the overuse of these antimicrobials has led to increasing antibiotic resistance. Consequently, alternative therapies, including plant extracts and probiotics, have been explored to reduce antibiotic dependency and mitigate resistance, especially in cases lacking veterinary oversight [5].

Despite these efforts, traditional approaches have certain limitations. Many traditional herbs chiefly restrict the growth of *Salmonella* in a single way [6,7,8,9]. For example, berberine present in Coptis chinensis mainly targets *Salmonella*’s type III secretion system [10]. However, these herbs often struggle to effectively restore the intestinal barrier through multiple pathways, which hinders the complete restoration of intestinal health during treatment. Additionally, some herbs exhibit potential toxic side effects at high doses. For instance, the extract of Tripterygium wilfordii may damage liver function at high doses, posing additional risks to the overall health of chicks [11].

Cinnamaldehyde (CA) is an aromatic aldehyde derived from the inner bark of the *Cinnamomum cinnamomi* plant. It is recognized by the U.S. Food and Drug Administration (FDA) as a Generally Recognized As Safe (GRAS) substance due to its ability to significantly enhance animal production performance, modulate immune function, and improve disease resistance [12,13]. Numerous studies have shown that CA has outstanding effects on animal husbandry. It can significantly improve the production performance of animals, effectively regulate immune function, and enhance disease resistance [14,15]. Additionally, recent studies indicate that the active aldehyde components of CA can interact with key immune cells, such as regulatory T cells, dendritic cells, macrophages, neutrophils, and B cells, thereby enhancing immune response [16,17,18]. CA can significantly enhance antioxidant enzyme activity in the intestine, thereby rapidly eliminating excessive free radicals in the body and minimizing oxidative stress damage to intestinal tissues [19,20]. However, limited research exists on the effects of CA on alleviating intestinal damage and improving gut microbial structure. Therefore, this study will investigate the beneficial effects of CA on intestinal tight junctions (TJs), intestinal microorganisms, and their metabolites in chicks infected with *S. pullorum*.

In this study, enzyme-linked immunosorbent assay (ELISA) was used to measure the expression levels of proinflammatory cytokines (IL-1β and TNF-α) and anti-inflammatory cytokines (IL-10) in the control group (CON), the *S. pullorum*-infected group (S.P), and the CA-treated group (CA) to evaluate the anti-inflammatory effects of CA. Histological analysis was conducted using H&E staining to assess the repair effects of CA on the intestinal tissue. Changes in intestinal tight junctions (TJs) were observed by quantifying the mRNA expression of TJ proteins (claudin-1, occludin-1, and zo-1) using RT-qPCR and ISH. Considering the critical role of the gut microbial composition in host nutrition, intestinal morphology, physiology, and immunity, 16S rRNA sequencing was performed to analyze microbial composition. Finally, targeted metabolomics analysis was employed to elucidate the effects of CA on intestinal function by characterizing metabolite profiles, quantifying their expression levels, and examining the correlations between specific metabolites and gut microbial composition.

## 2. Materials and Methods

### 2.1. Bacterial Strain and Drug Reagent

The experimental strain used was *S. pullorum* (CVCC1792), obtained from the National Veterinary Strain Collection Center (Beijing, China) and cultivated in trypticase soy broth (TSB) (Hopebio, Qingdao, China). All animal experiments were approved by the Animal Protection and Use Committee of Sichuan Agricultural University (approval number: 20230080). Cinnamaldehyde (>98% HPLC; CAS No. 104-55-2) was purchased from Chengdu Ruifensi Biotechnology Co. Ltd. (Chengdu, China).

### 2.2. Experimental Modeling and Grouping

**Figure 1:**
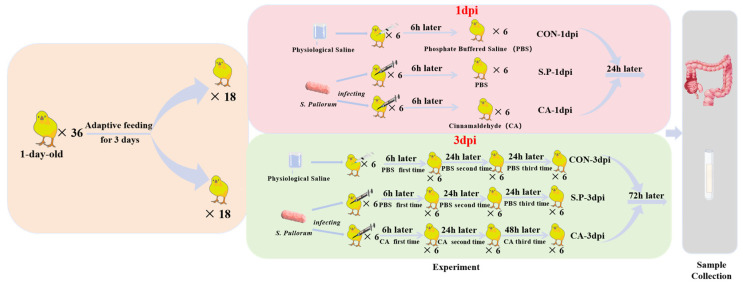
Experimental Flow Chart. A total of 36 chicks were grouped into 1 dpi and 3 dpi by time factor, a total of 18 chicks at each time point. Eighteen chicks from each time point were randomly divided into 3 groups, with the *S. pullorum*-infected and orally CA-treated group being the CA group. The group infected with *S. pullorum* without oral CA treatment was the S.P group. The group without *S. pullorum* infection was the CON group. Both 1 dpi and 3 dpi S.P and CA group chicks were infected with *S. pullorum* only once, the CA-1 dpi group received only one oral CA treatment, but the CA-3 dpi group received a total of 3 oral CA treatments.

One-day-old Salmonella-negative Hylandii Brown chicks (*n* = 36) were purchased from Chengdu Muxing Poultry Co., Ltd. (Chengdu, China). To prevent cross-infection, experimental animals from different groups were housed separately in the Veterinary Medicine, Sichuan Agricultural University animal facility. The chicks had free access to food and water. To minimize stress and ensure the accuracy of the experimental results, the chicks were acclimatized and fed for 3 days before experimental treatments. The body weight of the experimental chicks was approximately 55–60 g. An intraperitoneal injection of *S. pullorum* established a vertical infection model for pullorum disease. As shown in Figure 1, the study included 6 subgroups, each containing 6 chicks. The experiment was divided into two time points based on different sampling times after *S. pullorum* infection, i.e., 1 and 3 days post-infection (dpi). The S.P and CA groups were infected with *S. pullorum* at a concentration of 5 × 10^8^ CFU/mL, with a volume of 0.5 mL. The CON group was injected intraperitoneally with an equal volume of physiological saline. The therapeutic dose of CA was 100 mg/kg in the CA group, while the S.P and CON groups received an equal volume of phosphate-buffered saline (PBS) orally. The therapeutic dose of CA was determined based on our previous experiments, in which diseased chicks were treated with doses of 50 mg/kg, 100 mg/kg, and 150 mg/kg, respectively. The final results indicated that 100 mg/kg was the most scientifically appropriate. The specific data can be found in the Supplementary Materials. Samples were collected from experimental chicks at the 1 and 3 dpi time points, respectively, 24 h after the last CA treatment.

### 2.3. Sample Collection

Chicks were euthanized 1 and 3 days after *S. pullorum* infection, respectively. The ileum tissue and intestinal contents were carefully removed using scalpels and forceps. Intestinal samples were obtained from 18 chicks at 1 and 3 dpi, respectively. According to experimental requirements, the ileal tissue collected from each chick was divided into five intestinal segments of different lengths. These segments were used for H&E staining, biochemical analysis, in situ hybridization, RT-qPCR, and untargeted metabolomics. The intestinal contents were collected for 16S rRNA sequencing.

### 2.4. Histological Observations (H&E)

The intestinal tissue samples were immersed in 4% paraformaldehyde over 24 h, followed by dehydration and embedding in paraffin before histological analysis. Paraffin sections were stained with hematoxylin and eosin, dehydrated, sealed, and examined under a microscope.

### 2.5. Biochemical Analyses

Frozen intestinal tissue samples were thawed slowly on ice, ground into homogenate using a high-throughput tissue grinder (TL-48R, Shanghai Jingxin Industrial Development Co., Ltd., Shanghai, China), and then assayed for IL-1β, IL-10, and TNF-α according to the instructions of the kit (Jiangsu Meiman Industrial Co., Ltd., Yancheng, China).

### 2.6. In Situ Hybridization

The ileal tissue samples were fixed in in situ hybridization solution for over 24 h prior to the hybridization procedure, similar to the histological observation process. After fixation, the samples were dewaxed and rehydrated. Citric acid (pH 6.0) was used to facilitate tissue repair. Following natural cooling, proteinase K was added dropwise, and the samples were digested at 40 °C for 10 min. The samples were then rinsed with phosphate-buffered saline (PBS) three times and incubated with the prehybridization solution for one hour. Once this was completed, a hybridization solution containing the probe was added dropwise, and the samples were hybridized and washed. This procedure was repeated three times using the corresponding branching and signal-containing probe.

The nuclei were restained with DAPI staining solution, incubated for 8 min in the dark, rinsed, and sealed with an antifluorescence quenching sealer. Images were captured under a fluorescence microscope. Blue light: UV excitation wavelength 330–380 nm, emission wavelength 420 nm; green light: FAM (488) green excitation wavelength 465–495 nm, emission wavelength 515–555 nm; red light: CY3 red excitation wavelength 510–560 nm, emission wavelength 590 nm; pink light: CY5 excitation wavelength 608–648 nm, emission wavelength 672–712 nm. The nuclei of DAPI-stained cells appear blue, and positive expression is indicated by fluorescence labeling with the corresponding fluorophore, with intensity varying according to the expression level. In this experiment, yellow fluorescence indicates claudin-1 mRNA, green fluorescence indicates occludin-1 mRNA, and red fluorescence indicates zo-1 mRNA.

### 2.7. RT-qPCR

Frozen intestinal tissue samples were thawed slowly on ice and ground into homogenate with a high-throughput tissue grinder (TL-48R, Shanghai Jingxin Industrial Development Co., Ltd.) to detect the expression of target genes. Total RNA was extracted using TRIzol reagent (Biomedical Engineering Corporation, Beijing, China) according to the manufacturer’s instructions. RNA (5 μg/μL) was reverse transcribed into cDNA using the M-MLV 4 First-Strand cDNA Synthesis Kit (Biomedical Engineering Corporation, Beijing, China). Real-time fluorescence quantitative PCR was performed using Hieff UNICON^®^ Universal Blue qPCR SYBR Green Master Mix (Yeasen Biotechnology (Shanghai) Co. Ltd., Shanghai, China). The thermal cycling conditions were as follows: predenaturation at 95 °C for 2 min, followed by 40 cycles of denaturation at 95 °C for 19 s and extension at 60 °C for 30 s. Dissolution curves were plotted according to the instrument’s default settings. The internal reference gene used is β-actin, and the 2^−ΔΔCT^ method was used to calculate the relative expression levels of genes. The gene expression sequence primers (Youkang Biotechnology Co. Ltd., Hangzhou, China) are shown in Table 1.

### 2.8. The 16S rRNA Sequencing and Microbial Composition Analysis

Ultra-frozen intestinal content samples were sent to Shanghai Perrier Biotech (Shanghai, China) for microbial sequencing. Nucleic acids were extracted from the pretreated samples using the OMEGA DNA Kit (Omega Bio-Tek, Guangzhou, China). DNA content was determined using a NanoDrop (Thermo Scientific, Waltham, MA, USA). DNA fragments of approximately 480 bp were amplified by PCR, targeting the highly variable V3–V4 region of the bacterial 16S rRNA gene (primer sequence: ACTCCTACGGGGAGGCAGCA; reverse: GGACTACHVGGTWTCTAAT). Libraries were constructed using Illumina’s TruSeq Nano DNA LT Library Prep Kit and subjected to quality control and quantification. Microbial bioinformatics analysis was performed using QIIME2, including alpha and beta diversity analyses, and OTU classification using the Green genes database.

### 2.9. Untargeted Metabolomics

Ultra-frozen ileum tissue samples were transported to Shanghai Perrier Biotech (Shanghai, China) for untargeted metabolomics analysis using LC-MS. Briefly, the samples were thoroughly ground using a high-throughput tissue grinder (TL-48R, Shanghai Jingxin Industrial Development Co., Ltd., Shanghai, China). Each tissue sample (50 mg) was homogenized, sonicated with 800 μL of methanol (1:1, *v*/*v*) for 30 min, then frozen for 30 min and centrifuged at 4 °C, 12,000 rpm for 10 min. The supernatant was carefully collected, concentrated under vacuum until dry, and redissolved in 150 μL of 50% methanol (with 5 ppm L-2-chlorophenylalanine), then vortexed for 30 s. The precipitate was redissolved, centrifuged again (4 °C, 12,000 rpm, 10 min), and filtered through a 0.22 μm membrane. The filtrate was transferred to an assay bottle. To assess instrument stability and data reliability, 10 μL of each filtrate was mixed together to create a quality control (QC) sample. Chromatographic separation was performed on an ACQUITY UPLC HSS T3 column (100 Å, 1.8 μm, 2.1 mm × 100 mm) with a flow rate of 0.4 mL/min, column temperature of 40 °C, autosampler at 8 °C, and an injection volume of 2 μL. Positive ion mode was 0.1% formic acid, and negative ion mode was acetonitrile containing 0.1% formic acid. A Thermo Orbitrap Exploris 120 mass spectrometer using Xcalibur software (version 4.7) was used for data acquisition, and positive and negative ion modes both used DDA. The HESI source settings were 3.5 kV/3.0 kV spray voltage, 40 arb sheath gas, 15 arb auxiliary gas, capillary temperature of 325 °C, and auxiliary gas temperature of 300 °C. The primary resolution was set at 60,000, with a scan range of 100–1000 *m*/*z*, AGC target at standard, and max injection time at 100 ms. Secondary fragmentation was performed on the top 4 ions with an 8 s dynamic exclusion time, secondary resolution at 15,000, HCD collision energy of 30%, AGC target at standard. All data were processed in Compound Discoverer TM 3.3 software (version 3.3.2.31, Thermo, Waltham, MA, USA), where peak extraction, matching, and calibration were conducted using the software’s peak detection and quality scoring algorithms. Metabolites were identified by matching m/z values and standardized retention times against databases, with annotations based on the HMDB and Kyoto Encyclopedia of Genes and Genomes (KEGG).

To identify metabolites that differed between groups, multivariate statistical analyses, including principal component analysis (PCA) and partial least squares discriminant analysis (PLS-DA), were performed, along with univariate analyses (fold change (FC) and Student’s *t*-test). Data were further analyzed using clustering, log2 transformation, and z-score normalization (zero mean normalization). Hierarchical clustering was performed using Euclidean distance to assess the metabolic pathway enrichment of differential metabolites in the KEGG database. Pathways with *p* < 0.05 were considered significantly enriched for differential metabolites.

### 2.10. Correlation Analysis Between the Microbiome and Metabolome

We compared the top 50 most abundant microbiome data obtained from 16S rRNA sequencing with the top 50 metabolite data obtained from untargeted metabolomics (LC-MS) assays using Spearman correlation analyses to assess the correlation between metabolite intensity and microbial abundance.

### 2.11. Statistical Analysis

IBM SPSS Statistics (R26.0.0.0, Inc., Chicago, IL, USA) was used to perform ANOVA and Student’s *t*-tests on all data. All experimental data are expressed as mean ± standard error of the mean (S.E.M.), with *p* < 0.05 considered statistically significant.

## 3. Results

### 3.1. CA Protects Against Pullorum Disease by Restoring Intestinal Tight Junctions

**Figure 2:**
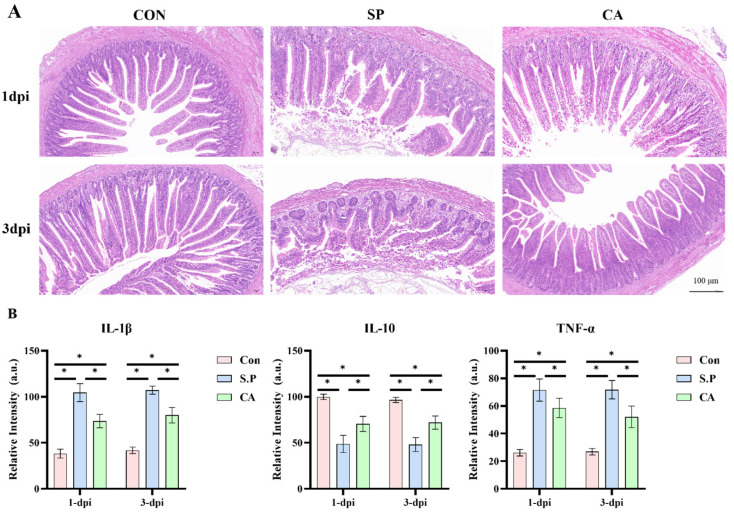
(**A**) Representative intestinal pathology images of the H&E staining, scale bar: 100 μm. (**B**) Effects of cinnamaldehyde on the expression of IL-1β, IL-10, and TNF-α in intestinal tissues (*p* < 0.05). CON: control group; SP: *S. pullorum* infection group; CA: CA treatment group. * *p* < 0.05.

Regarding clinical manifestations, the chicks in each group exhibited different symptoms. Chicks in the S.P group showed loss of appetite and exhibited piling behavior, depressed spirit, loose fluff, diarrhea, and white fecal slime visible at the anus. In contrast, chicks in the CA group showed noticeable improvement on the second and third days after gavage administration, with increased vigor and reduced dysentery. The CA group showed the proinflammatory cytokines IL-1β and TNF-α in ileal tissues, which were significantly higher in the S.P group. However, the expression was significantly decreased in the CA group (*p* < 0.05). Meanwhile, the expression of the anti-inflammatory cytokine IL-10 showed an opposite trend to that of proinflammatory factors, with a significant decrease in the S.P group compared to the CON group and there was an increase in IL-10 levels after treatment with CA (*p* < 0.05) on the second and third day after gavage administration, with a slight increase in vigor and an improvement in dysentery. Histopathological examination (H&E staining) revealed that the ileal tissue in the CON group was regularly arranged and densely organized without apparent lesions. The intestinal villi of the S.P group were broken and irregularly arranged, the epithelial cells were detached, and the ileal histomorphology was significantly improved in the CA group compared to the S.P group (Figure 2A). Following infection with *S. pullorum*, immune cells respond to the bacteria by secreting various cytokines. Our research revealed that the expression of proinflammatory cytokines IL-1β and TNF-α in ileal tissues was significantly elevated in the S.P group. At the same time, it notably decreased in the CA group (*p* < 0.05). In contrast, the expression of the anti-inflammatory cytokine IL-10 was significantly lower in the S.P group compared to the CON group. However, it showed an increase after CA treatment (*p* < 0.05) (Figure 2B).

**Figure 3:**
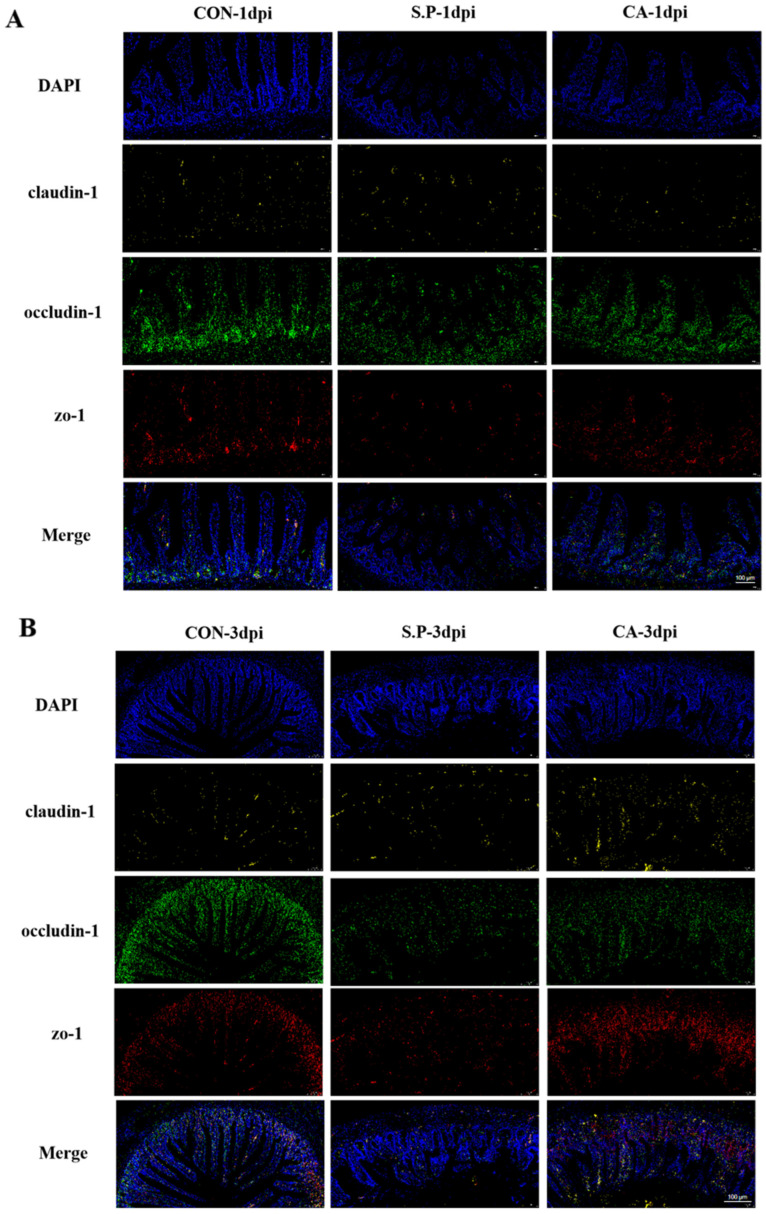

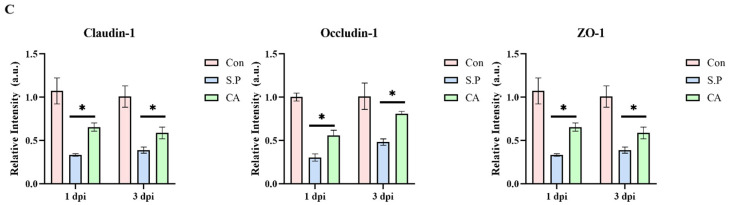
Effectiveness of CA in repairing intestinal TJ damage. (**A**) Fluorescence intensity representation of intestinal TJ protein mRNA expression at 1 dpi; scale bar: 100 μm. (**B**) Fluorescence intensity representation of intestinal TJ protein mRNA expression at 3 dpi; scale bar: 100 μm. (**C**) Real-time fluorescence quantification of mRNA expression of ZO-1, Occludin, and Claudin-1 in the intestinal tissues (*p* < 0.05). * *p* < 0.05.

It is well established that intestinal TJs play a primary role in preventing the invasive process of *S. pullorum*. We used in situ hybridization (ISH) and RT-qPCR to examine the role of CA in restoring intestinal TJs. As shown in Figure 3A,B, the fluorescence intensity indicating gene expression in the S.P group was weaker than in the CON group, while it was stronger in the CA group. RT-qPCR results showed that the mRNA expression of the three proteins was significantly decreased in the S.P group compared to the CON group and up-regulated in the CA group compared to the S.P group (*p* < 0.05) (Figure 3C).

### 3.2. CA Affects the Structure of the Gut Microbial Composition

**Figure 4:**
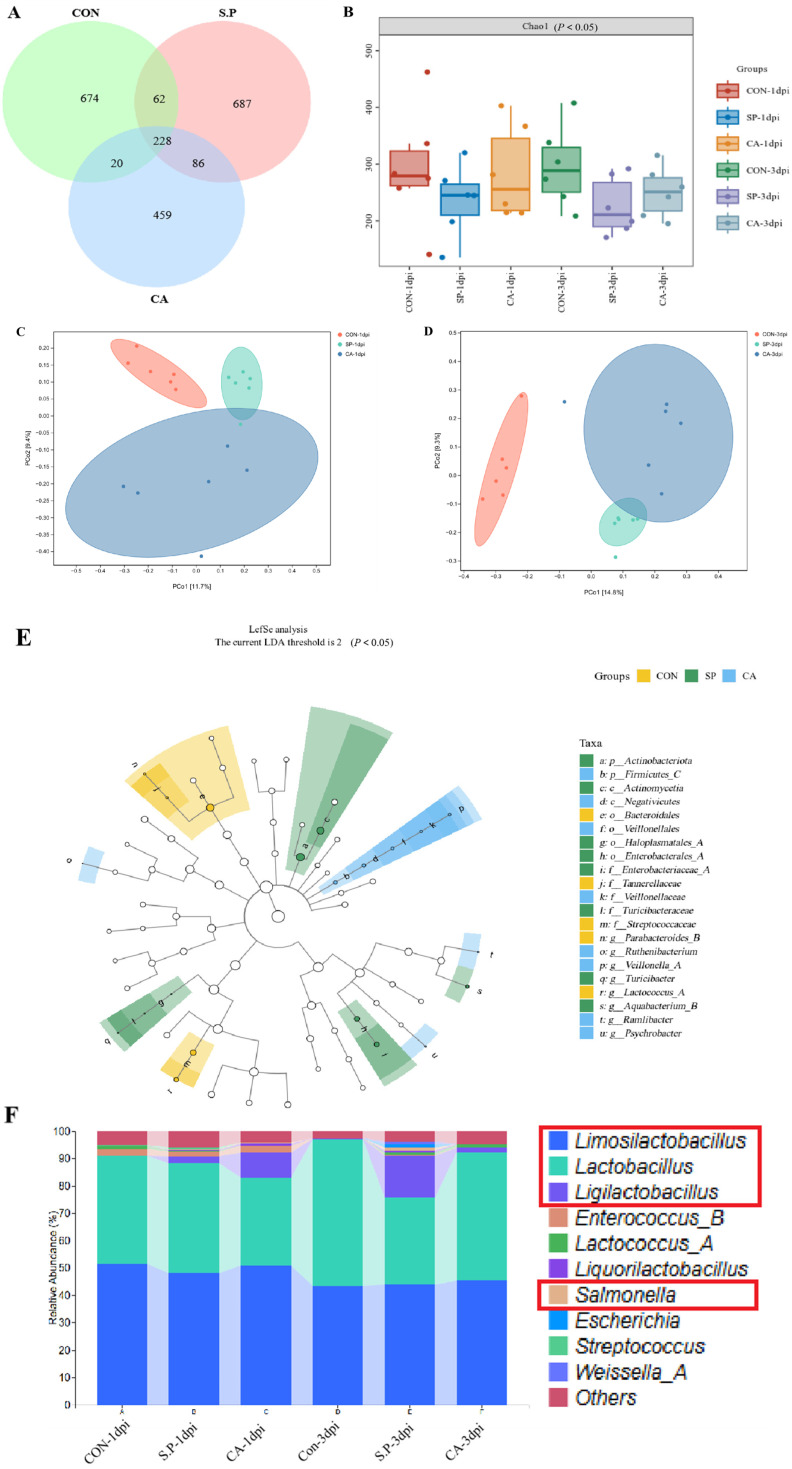
CA reverses gut microbial disorders caused by *S. pullorum* infection. (**A**) Venn diagram showing the overlap of OTUs found in the gut microbiota of the CON, S.P, CA groups; (**B**) Alpha diversity index (*p* < 0.05); (**C**,**D**) Principal Coordinate Analysis (PCOA) of the microbiota based on Bray–Curtis metrics; (**E**) LefSe analysis (the current LDA threshold is 2) (*p* < 0.05); (**F**) Differential microorganisms in the top 10 most abundant (Genus level). “Others” represent the minor bacterial families whose relative abundances were  <0.1%.

To determine whether the beneficial effect of CA is due to improvements in the structure of the chick gut microbial composition, we performed 16S rRNA gene sequencing. In total, 2216 OTUs were identified, with 228 OTUs shared across the three experimental groups. There were 674, 687, and 459 unique OTUs in the CON group, S.P group, and CA group, respectively (Figure 4A). Gut microbial alpha diversity was significantly reduced after *S. pullorum* infection compared to the CON group (*p* < 0.05). Meanwhile, the CA group showed an inevitable upward trend compared to the S.P group (*p* < 0.05). The results at 3 dpi follow the same trend as the results at 1 dpi (Figure 4B). Principal coordinate analysis (based on the Jaccard distance algorithm) showed separation in gut microbial composition between the CON-1 dpi group and the S.P-1 dpi group, with a small area of overlap between the S.P-1 dpi group and the CA-1 dpi group. Similarly, separation in gut microbial composition was observed between the CON-3 dpi group and the S.P-3 dpi group, with a slight overlap between the S.P-3 dpi group and the CA-3 dpi group (Figure 4C,D). LefSe analysis shows significant differences in bacterial characteristics among the three groups. The threshold for LDA was set to 2, which allowed the identification of taxa with significant differences in relative abundance. In the CON group, *Streptococcaceae* and *Ligilactobacillus* were significantly enriched, with an LDA score over 3.5, indicating that these taxa are more abundant in the baseline uninfected state (*p* < 0.05). In the S.P group, *Bacteroidales*, *Tannerellaceae, Parabacteroides*, and *Actinomycetia* had significant enrichment, with LDA scores ranging from 2.5 to 3.5 (*p* < 0.05). In comparison, there is an increased relative abundance of *Veillonellales*, *Fusobacterium*, *Psychrobacter*, and *Ruminiclostridium* in the CA group, with LDA scores between 2 and 3 (*p* < 0.05) (Figure 4E). At the genus level, the results of the relative abundance analyses showed different microbial compositions in different groups. The S.P group decreased in *Limosilactobacillus* and *Lactobacillus*, while *Salmonella* abundance increased significantly. After CA treatment, *Salmonella* decreased, and *Ligilactobacillus* significantly increased; *Lactobacillus* largely recovered (Figure 4F).

### 3.3. CA Improves S. pullorum-Induced Intestinal Metabolic Dysfunction

**Figure 5:**
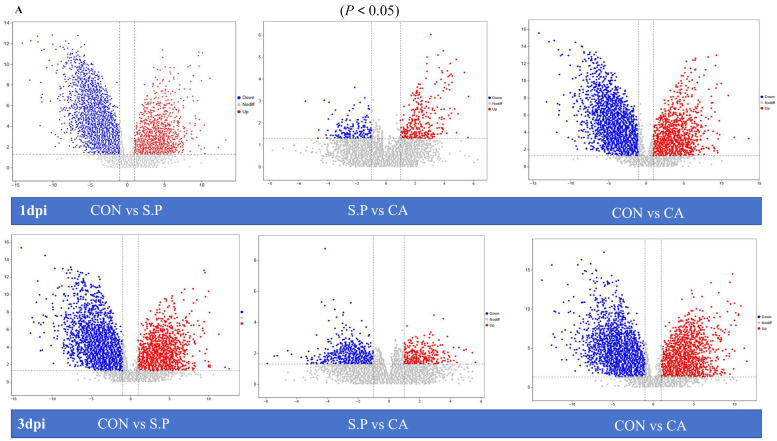

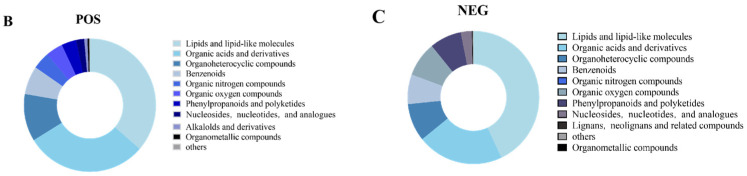

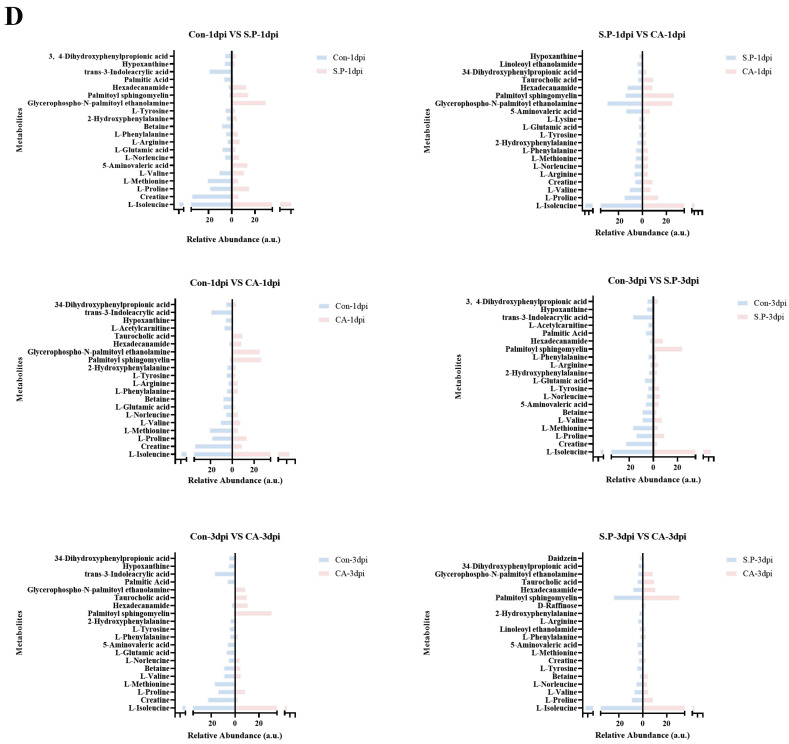

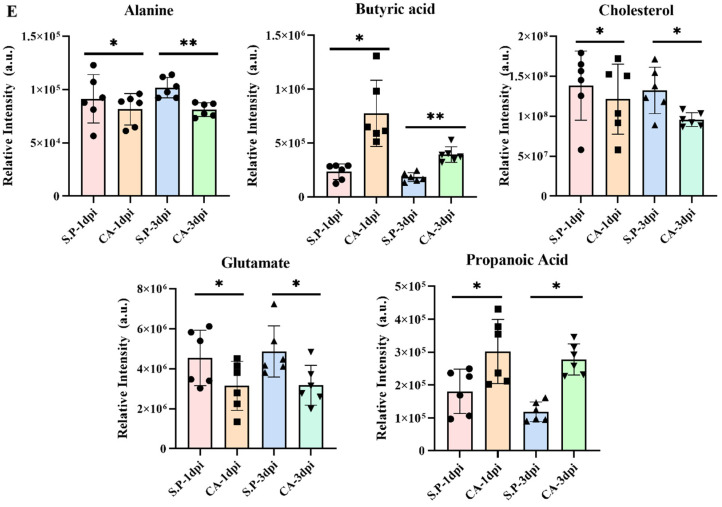
CA alters intestinal metabolic disorders due to *S. pullorum* infection. (**A**) Volcano plot illustrating the up-regulation and down-regulation of genes in pairwise comparisons among different groups. In the plot, red dots represent up-regulated genes, blue dots denote down-regulated genes, and grey dots indicate genes with no significant difference; (**B**) Pie chart of metabolite enrichment in POS mode (top 10); (**C**) Pie chart of metabolite enrichment in NEG mode (top 10); (**D**) Comparison of top 20 metabolite abundance (*p* < 0.05); (**E**) Histogram of alanine, butyric acid, cholesterol, glutamate, and propanoic acid, 5 metabolites in the S.P and CA groups. * *p* < 0.05, ** *p*< 0.01.

Changes in the structure of gut microbes will inevitably lead to alterations in metabolism. CA (presumably a treatment) promotes the healing of diseases by influencing intestinal metabolism. Figure 5A illustrates a volcano plot depicting differential metabolite variations across three groups (*p* < 0.05). At 1 dpi, 697 distinct metabolites were identified in the metabolite analysis comparing the CON group and the S.P group, which included 252 metabolites that were up-regulated and 445 that were down-regulated. When comparing the CON group to the CA group, 755 differential metabolites were identified, consisting of 268 up-regulated and 487 down-regulated metabolites. Between the S.P and CA groups, 75 variable metabolites were characterized, including 43 that were up-regulated and 32 that were down-regulated. Similarly, the volcano plot for 3 dpi shows that 739 differential metabolites were identified between the CON and S.P groups, which included 325 up-regulated and 414 down-regulated metabolites. Additionally, 730 metabolites exhibited differential regulation between the CON and CA groups, comprising 303 up-regulated and 427 down-regulated metabolites. There were 125 differential metabolites identified between the S.P and CA groups, consisting of 66 that were up-regulated and 89 that were down-regulated. Figure 5B,C display the top 10 metabolites detected in ileal tissue. Metabolite analysis in both positive and negative modes revealed that lipids and lipid-like molecules, along with organic acids and their derivatives, dominate the gut metabolite profile, accounting for 36.4% and 29.8% in positive mode and 43.0% and 21.2% in negative mode, respectively. We then compared the differences in the abundance of the top 20 metabolites across different groups (Figure 5D). The expression of the same metabolites varies between groups. Moreover, the top 20 metabolites were enriched differently in each pairwise comparison group, indicating that intestinal metabolism undergoes considerable changes after *S. pullorum* infection and that intestinal metabolism is restored following CA treatment. For instance, in the comparison between the CON and S.P groups, creatine expression was significantly higher in the CON group than in the S.P group. In the comparison between the S.P and CA groups, creatine levels were increased in the CA group compared to the S.P group (*p* < 0.05). To gain a more targeted understanding of the effects of *S. pullorum* infection on host metabolism, five metabolites—alanine, butyric acid, cholesterol, glutamate, and propanoic acid—were explicitly selected to assess the differences in their levels between the CON and S.P groups (Figure 5E). The results indicated that *S. pullorum* infection increased the amount of butyric acid while alanine, cholesterol, glutamate, and propanoic acid levels decreased (*p* < 0.05).

**Figure 6:**
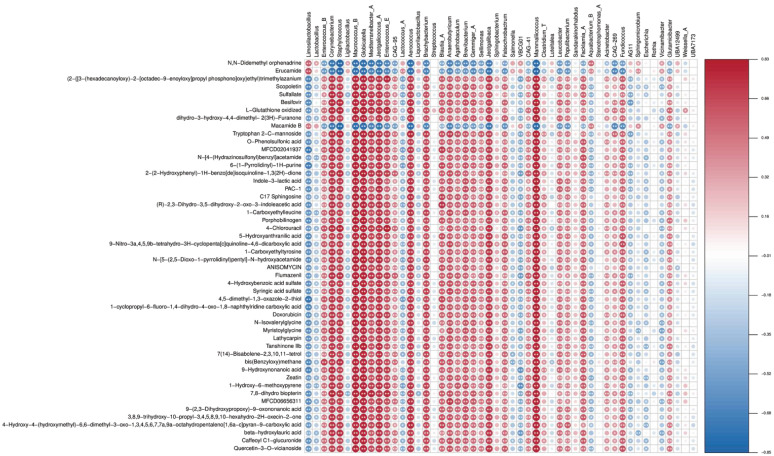
Gut microbiome–metabolome correlation analysis. The depth of colors ranging from blue to red represents the magnitude of correlation. * *p* < 0.05, ** *p* < 0.01.

We assessed the gut microbiome and metabolome relationship using Spearman correlation and linear regression. As shown in Figure 6, *Corynebacterium*, *Staphylococcus*, *Macrococcus B*, *Globicatella*, *Jeotgalicoccus A*, *Aerococcus*, *Brachybacterium*, *Jeotgalibaca*, and *Mammaliicoccus* exhibited the highest number of positive correlations, each associated with 47 metabolites, while *Mediterraneibacter A* showed positive associations with 45 metabolites. Other taxa, including *Corynebacterium*, *Staphylococcus*, *Macrococcus B*, *Globicatella*, *Mediterraneibacter A*, *Jeotgalicoccus A*, *Enterococcus E*, *Aerococcus*, and *Brachybacterium*, each showed negative associations with three groups metabolites. For instance, *Limosilactobacillus* was negatively associated with O-phenolsulfonic acid. *Mediterraneibacter A* was negatively associated with indole-3-lactic acid and *Enterococcus E* with PAC-1.

## 4. Discussion

PD, caused by *S. pullorum*, is a disease that can spread both horizontally and vertically. It is well established that some chicks infected during early life who survive clinical disease may not exhibit overt signs of infection but can become carriers. In adult chickens, intestinal carriage of *S. pullorum* does not result in severe gastrointestinal disease, but it is fatal for chicks [21]. In the context of antidrug reduction and replacement, there is an urgent need to develop new drugs to alleviate the drug resistance challenge. Herbal medicines are typically natural, green, and organic and, unlike commonly used antibiotics, herbal compounds exhibit minimal toxicity and reduced potential for drug resistance, even with prolonged use. They offer disease prevention and treatment benefits while simultaneously enhancing poultry productivity and modulating immune responses [22]. As a traditional herbal remedy, cinnamon demonstrates inherent antibacterial, anti-inflammatory, and antioxidant properties primarily due to CA [15]. Research indicates that CA can effectively inhibit foodborne pathogens, with demonstrated pharmacological activities including anti-inflammatory, anticancer, antiulcer, and antidiabetic effects [16,23,24,25]. Additionally, CA has been shown to promote colon health, improve blood circulation, and stimulate tissue regeneration, making it a promising candidate for treating pullorum disease [26]. In this experimental study, we systematically and comprehensively investigated the effects of CA on controlling pullorum disease in chicks by examining its impact on three key areas: intestinal TJs, gut microorganisms, and intestinal metabolism. Although the therapeutic effects of CA may vary among individuals, overall, our study observed significant therapeutic effects. This multifaceted approach aims to better understand CA’s therapeutic potential in enhancing poultry health and disease resistance.

*S. pullorum*-infected chicks often show distinct clinical signs of disease. *S. pullorum* initially evades the body’s immune response to establish infection, followed by activation of intestinal epithelial cells and recruitment of macrophages, neutrophils, and eosinophils into the intestinal lumen [27]. These immune cells secrete more proinflammatory cytokines and reduce the release of anti-inflammatory factors, triggering a localized inflammatory response. The results of our experiments confirm this, as levels of IL-1β and TNF-α were significantly increased in intestinal tissues after *S. pullorum* infection, while IL-10 levels were significantly lower [28]. However, it is encouraging to note that proinflammatory cytokine levels were lower and anti-inflammatory cytokine levels were higher after treatment with CA (*p* < 0.05). TJ proteins are essential components that form tight junctions (TJs) between intestinal epithelial cells. Changes in their expression can directly impact the integrity of the intestinal barrier. TJs serve to prevent harmful substances such as bacteria, toxins, and antigens in the intestinal lumen from entering the body’s internal tissues. When chicks are infected with *S. pullorum*, the structure of these tight junctions is disrupted, resulting in increased intestinal permeability. This disruption makes it easier for pathogens like *Salmonella* to penetrate the intestinal epithelium and enter the bloodstream and surrounding tissues, leading to systemic infection and disease [29]. Our study revealed that the mRNA expression levels of TJ proteins—specifically occludin-1, claudin-1, and zo-1—were significantly reduced in chicks infected with *S. pullorum*. This finding suggests that alterations in the expression of these TJ proteins are directly linked to a compromised intestinal barrier function. In addition to maintaining barrier integrity, TJ proteins also play a role in intracellular signal transduction and immune regulation. Variations in their expression can activate or inhibit various signaling pathways, subsequently impacting cellular behaviors such as proliferation, differentiation, and apoptosis. Our research indicates that the down-regulation of TJ protein mRNA is associated with an up-regulation of proinflammatory cytokines in the intestine, including TNF-α. This suggests that changes in TJ protein expression affect the intestinal immune microenvironment and contribute to the host immune response against *S. pullorum* infection. Furthermore, we observed increased TJ protein mRNA expression following treatment with CA. A variety of proteins play a role in the composition of TJs, and based on their different roles, these proteins can be categorized as structural and regulatory proteins [30,31]. Damage to occludins, claudins, and ZOs disrupts the intestinal barrier function and increases mucosal permeability [30,32]. Our results suggest that *S. pullorum* infection disrupts TJs (e.g., zo-1, occludin-1, and claudin-1) between intestinal epithelial cells and increases intestinal permeability. In addition, CA enhances the recovery of TJ proteins mRNA (zo-1, occludin-1, and claudin-1) and repairs the intestinal villi, thus promoting the recovery of intestinal barrier function.

Additionally, poultry gut health depends on a balance between the immune system and the microbial composition [33]. The relative stability of the type and quantity of intestinal flora serves as a key indicator for assessing host health. Our study revealed that *S. pullorum* infection disrupts gut microbiota, significantly reducing in alpha diversity, indicating a decrease in gut microbial species. Compared to 1 dpi, the situation at 3 dpi suggests that *S. pullorum* infection may result in gut dysfunction. However, treatment with CA increased alpha diversity at 1 dpi and 3 dpi, indicating that CA effectively modulates the diversity of the gut flora. E Van [34] found that CA can, in the intestinal tract of pigs, promote the reproduction and growth of beneficial bacteria and inhibit the reproduction of harmful bacteria to regulate intestinal flora and enhance immunity. Our results show that lactic acid bacteria showed the most significant increase in abundance among all microbial groups. This increase is likely due to CA treatment, which promotes the establishment of dominant gut microbes, enhances chick resistance to *Salmonella*, and reduces *Salmonella* counts while increasing lactic acid bacteria abundance. Beta diversity analysis indicated that CA treatment resulted in an overlap of gut microbial communities in infected chicks at 1 dpi and 3 dpi post-infection, with the overlap being more pronounced at 3 dpi. This suggests that CA treatment was less effective at restoring gut microbial balance at 3 than 1 dpi. One possible explanation for this observation is the less severe gut disruption caused by *S. pullorum* during the early stages of infection, which may allow for more effective treatment in these initial phases. Our findings align with E Van’s, reinforcing the idea that CA can effectively restore the balance of gut microbiota.

LEfSe analysis identified *Lactococcus*, *Bacteroidetes*, *Streptococcaceae*, *Tannerellaceae*, and *Parabacteroides* as the dominant species in healthy chicks. Notably, *Bacteroidetes*, *Tannerellaceae*, and *Parabacteroides* are known to produce short-chain fatty acids (such as acetate and butyrate) through carbohydrate fermentation, which support immune responses and promote gut health. In contrast, *Lactococcus* and *Streptococcaceae* regulate the gut’s acidic environment via lactic acid fermentation, inhibiting pathogen growth and enhancing gut immunity. In the S.P group, we observed a significant increase in pathogenic bacteria, such as *Enterobacterales*, indicating that *S. pullorum* infection led to gut microbial dysbiosis. Our findings suggest that *S. pullorum* can elicit inflammatory responses by altering gut microbial composition, consistent with previous experimental results. However, the CA group exhibited inhibitory effects on these harmful bacteria and showed significant enrichment of *Veillonella* and *Psychrobacter*, which possess anti-inflammatory and antimicrobial properties. This suggests that CA may ameliorate infection-induced dysbiosis by modulating gut microbial populations.

The alterations in the microbiota have a significant impact on intestinal health and host disease resistance. The impact on intestinal health is evident in enhancing both the intestinal barrier and immune functions. After treatment with CA, there was a significant increase in the abundance of beneficial gut bacteria, particularly *Lactobacillus*. These beneficial microorganisms promote the growth and repair of intestinal epithelial cells in poultry and enhance the expression of TJ proteins. This process aids in restoring the gut’s physical barrier function, thereby preventing the entry of pathogens and harmful substances into the body [35]. Beneficial microbes also activate gut immune cells, enhancing immune responses and regulating gut immune factor secretion [36]. Changes in the microbiota also affect the secretion of immune factors in the gut. Our research observed that, after treatment with CA, the levels of proinflammatory cytokines such as IL-1β, TNF-α, and IL-10 in the gut were regulated, thus avoiding excessive inflammatory responses. The impact of microbial changes on host disease resistance is mainly manifested in the inhibition of pathogen growth and the enhancement of the host’s overall disease resistance [37]. The restored beneficial microbial community can compete with pathogens for nutrients and ecological niches. For instance, beneficial bacteria such as *Lactobacillus* can proliferate extensively in the gut, occupying the adhesion sites on the intestinal mucosal surface, thereby preventing pathogens like *Salmonella* from adhering and growing. The normal intestinal microbiota contributes to immune memory formation by the immune system. After treatment with CA, the intestinal microbiota is restored to balance, enabling the immune system to more rapidly and effectively initiate an immune response to diseases. The balance of the gut microbiota is closely related to the host’s metabolic functions. The increase in beneficial microbes can promote the digestion and absorption of nutrients, thereby improving the health status of chicks [38]. Good nutritional status and health conditions help chicks maintain a robust immune system, thus enhancing overall disease resistance. In our results, CA treatment improves gut microbiology in the intestinal environment in chicks, as indicated by the preservation of villus morphology and reduction in pathogenic signals. The increase in beneficial bacteria such as *Limosilactobacillus* and *Lactobacillus*, combined with a reduction in pathogenic *Salmonella* levels, illustrates CA’s dual antimicrobial and probiotic-promoting properties. This gut microbial shift is essential for maintaining a functional microbial barrier, which, in turn, supports the immune system in managing pathogenic challenges.

The composition and function of biomes are inseparable, and we have revealed the composition of the gut microflora through 16s RNA but were unable to provide information on host–microbe interactions. Therefore, we used untargeted metabolomics to further analyze the dynamics of intestinal species metabolites in response to changes in the metabolic function of the microbial community to host infection by pullorum disease and in response to CA treatment. From the metabolite species chart, lipids and lipid-like molecules (e.g., phospholipids and cholesterol) and organic acids and molecules (e.g., short-chain fatty acids) make up more than half. Lipid metabolites are often involved in physiological processes such as cell membrane construction, signaling, and energy storage and also play a crucial role in regulating the physical barrier function of the intestinal tract and mitigating inflammatory responses [39,40,41,42]; organic acids and their derivatives, as metabolites in the fermentation process of intestinal microorganisms, can supply energy to microorganisms, regulate the intestinal microenvironment, and inhibit the growth of pathogens [43,44,45]. Two-by-two comparisons through volcano diagrams showed that CON vs. S.P had a higher total number at 3 dpi in both the 1 dpi and 3 dpi cases, suggesting that the complexity of their infections increased with time. On the other hand, the number of metabolic differentiators in S.P vs. CA at 1 dpi was doubled compared to 3 dpi. And the total number of CON vs. CA differential metabolites was higher at 3 dpi than at 1 dpi, suggesting that using CA may be more effective in freshly infected fowl with white diarrhea. Finding the key metabolites is essential to understand the mechanism of CA treatment of pullorum disease. We found that amino acids such as L-isoleucine, L-proline, L-valine, and L-arginine were higher in the S.P group than in the CA group. The reason for this may be that, due to the stress response of the body caused by infection with *S. pullorum*, its immune requirements increase, the body’s proteolysis is accelerated, and more amino acids are needed for coping with acute phase protein deficiencies [46,47]. Palmitoyl sphingomyelin and taurocholic acid content was low in the S.P group and high in the CA group, which may be due to the following reasons. Upon infection with *S. pullorum*, there is an upsurge in intracellular sphingomyelinase activity, which triggers the hydrolysis of palmitoyl sphingomyelin. Concurrently, this bacterium impairs the function of the intestinal barrier. When fatty acid synthesis and catabolism are disrupted, the levels of taurine-conjugated bile acids, such as taurocholic acid, experience a significant decline [48,49].

Within the CA group, we observed a deceleration of the inflammatory process, accompanied by a restoration of the synthesis and metabolism of palmitoyl sphingomyelin and taurine-conjugated bile acid. Our analysis indicates that certain amino acids, cholesterol, and short-chain fatty acids play a moderately significant role in the host’s metabolism. Consequently, we chose to focus on the alterations in butyric acid, alanine, glutamate, cholesterol, and propanoic acid to depict the in vivo metabolic processes of the host. Both butyric acid and propanoic acid reduce the expression of proinflammatory factors and inhibit the growth of *Salmonella* spp. within the host [50]. It can be obtained from the 1 dpi and 3 dpi data that CA promotes butyric acid and propanoic acid and is higher at 1 dpi, suggesting that early treatment of pullorum disease may be better. Glutamate and alanine have been shown to provide metabolic energy to intestinal cells and enhance intestinal immune cell activity [51,52,53]. Decreased levels of glutamate and alanine after CA treatment proved that the host organism is undergoing repair, consuming amino acids, and synthesizing proteins. Cholesterol has been shown to play a crucial role in enhancing cell membrane stability, promoting bile acid absorption, and maintaining intestinal ecological balance [54,55]. The reduced cholesterol levels following CA treatment may be attributed to the enhanced absorption of bile acids. Conversely, they could also be linked to the action of Chaihu saponin A, which shares a similar mechanism that disrupts the formation of lipid rafts and interferes with actin rearrangement caused by *S. pullorum*. These key metabolites exhibit complex and interconnected relationships, collaborating to support the recovery of chicks’ health. For instance, butyrate can influence lipid metabolism in the liver by activating specific signaling pathways and inhibiting cholesterol synthesis [56]. When CA increases the butyrate concentration in the gut, it sends signals to the liver to reduce the activity of enzymes involved in cholesterol synthesis, thereby decreasing cholesterol levels. This helps to alleviate the metabolic burden on the liver under infection stress. In addition, butyrate provides energy to intestinal epithelial cells and promotes their repair and regeneration, affecting amino acid metabolism in the gut. As the function of intestinal epithelial cells gradually returns to normal, the abnormal demand for amino acids such as glutamate and alanine decreases, resulting in reduced levels of these amino acids in the gut [57]. Butyrate possesses anti-inflammatory properties that aid in reducing intestinal inflammation. During inflammatory stress, the amino acids glutamate and alanine contribute to immune responses and adaptations in energy metabolism, leading to elevated levels of these amino acids [58,59]. The relationship between changes in gut microbiota and metabolic alterations is bidirectional. For instance, the gut microbiota metabolizes amino acids like glutamate and alanine, subsequently influencing growth and metabolism. When the levels of glutamate and alanine diminish, this shift can alter the nutritional environment for the gut microbiota, impacting the growth and metabolism of propionate-producing bacteria and decreasing propionate levels [60,61]. The interactions among these key metabolites are interdependent and regulatory, creating a complex metabolic network. CA controls the levels of these metabolites within the network, working together to enhance the recovery of the intestinal tract in chicks and improve their overall health.

Gut microbes can produce various active metabolites. Changes in gut microbial diversity will inevitably alter the molecular composition of these metabolites [62,63,64]. Positive correlations have been identified between specific taxa, such as Corynebacterium (including *Erucamide*), and metabolites like mustard amide, which play roles in immune and inflammatory responses. This observation suggests that CA restores microbial diversity and promotes beneficial interactions within the microbiome–metabolome axis. In contrast, negative correlations, such as the one between *Limosilactobacillus* and O-phenolsulfonic acid, may indicate competitive or degradative processes contributing to homeostasis. The capacity of CA to selectively modulate the gut microbiota and the associated metabolic network underscores its potential as a versatile therapeutic agent for enhancing poultry health. The application of CA in commercial poultry production is reinforced by its positive effects on intestinal health and its ability to improve disease resistance in chicks. On the one hand, CA reduces the risk of infections in poultry, lowers disease-related mortality rates, and enhances breeding efficiency. On the other hand, its use decreases the reliance on antibiotics, mitigates the risk of antibiotic residues, and aligns with the growing trend toward antibiotic-free (ABF) poultry farming.

## 5. Conclusions

In conclusion, this study underscores the multifaceted effects of CA in treating pullorum disease. By restoring intestinal TJs, enhancing immune function, rebalancing microbial composition, and reprogramming metabolism, CA demonstrates the potential for improving immune resilience and metabolic adaptation in chicks. However, further research is essential to determine whether CA can be a viable alternative to antibiotics. Future studies should focus on elucidating the specific molecular mechanisms behind CA’s influence on these interconnected systems and evaluating its effectiveness under various environmental conditions. Additionally, it will be important to investigate the long-term effects, safety, and practical applications of CA in commercial poultry settings.

## Figures and Tables

**Table 1:** Primer sequence.

Gene Name	Primer (5′–3′)
*β-actin* F	CCAAAGCCAACAGAGAGAAG
*β-actin* R	ACACCATCACCAGAGTCCA
*Claudin-1* F	GAAGCATTTGGAGAGGACAC
*Claudin-1* R	GCTGGGAGAGAAAGGAGAA
*Occludin-1* F	CTGCTCTGCCTCATCTGCTT
*Occludin-1* R	GTTCTTCACCCACTCCTCCAC
*zo-1* F	GTTTGTATGGCTTTTGTGCTT
*zo-1* R	TTCCTCTGGTTCTCTTCTGG

## Data Availability

The raw data supporting the conclusions of this article will be made available by the authors, without reservation.

## Supplementary Materials

The following supporting information can be downloaded at: https://www.mdpi.com/article/10.3390/vetsci12030237/s1, Figure S1: The daily survival rate of chicks from 1 to 5 days in different group. Table S1: Clinical behavior observation grading table. Table S2: Clinical behavior of chicks in each group.

## References

[1] Kang X., Jin C., Gu P., Wang S., Gao Z., Dai C., Zhou X., Siddique A., Zhou H., Huang L. (2024). A dynamic platform for global pullorum disease and fowl typhoid. Anim. Dis..

[2] Chen J., Zhou X., Tang Y., Jiang Z., Kang X., Wang J., Yue M. (2023). Characterization of two-component system CitB family in Salmonella enterica serovar Gallinarum biovar Gallinarum. Vet. Microbiol..

[3] Farhat M., Khayi S., Berrada J., Mouahid M., Ameur N., El-Adawy H., Fellahi S. (2023). Salmonella enterica Serovar Gallinarum Biovars Pullorum and Gallinarum in Poultry: Review of Pathogenesis, Antibiotic Resistance, Diagnosis and Control in the Genomic Era. Antibiotics.

[4] Julianingsih D. (2023). Causative Agents for Fowl Typhoid and Pullorum Disease in Poultry and Approach to Control. Master’s Thesis.

[5] de la Fuente-Nunez C., Cesaro A., Hancock R.E. (2023). Antibiotic failure: Beyond antimicrobial resistance. Drug Resist. Updates.

[6] Dhanda G., Acharya Y., Haldar J. (2023). Antibiotic adjuvants: A versatile approach to combat antibiotic resistance. ACS Omega.

[7] Rabetafika H., Razafindralambo A., Ebenso B., Razafindralambo H. (2023). Probiotics as Antibiotic Alternatives for Human and Animal Applications. Encyclopedia.

[8] Wang J., Deng L., Chen M., Che Y., Li L., Zhu L., Chen G., Feng T. (2024). Phytogenic feed additives as natural antibiotic alternatives in animal health and production: A review of the literature of the last decade. Anim. Nutr..

[9] Ayalew H., Zhang H., Wang J., Wu S., Qiu K., Qi G., Tekeste A., Wassie T., Chanie D. (2022). Potential feed additives as antibiotic alternatives in broiler production. Front. Vet. Sci..

[10] Yang L., Sun J., Yang T., Zhang X., Xu C., Wei Y., Li Y., Zhao Y., Zhang S., Wu Q. (2024). Therapeutic effects and mechanisms of berberine on enteritis caused by Salmonella in poultry. Front. Microbiol..

[11] Cui D., Xu D., Yue S., Yan C., Liu W., Fu R., Ma W., Tang Y. (2023). Recent advances in the pharmacological applications and liver toxicity of triptolide. Chem. Biol. Interact..

[12] Shu C., Ge L., Li Z., Chen B., Liao S., Lu L., Wu Q., Jiang X., An Y., Wang Z. (2024). Antibacterial activity of cinnamon essential oil and its main component of cinnamaldehyde and the underlying mechanism. Front. Pharmacol..

[13] Guo J., Yan S., Jiang X., Su Z., Zhang F., Xie J., Hao E., Yao C. (2024). Advances in pharmacological effects and mechanism of action of cinnamaldehyde. Front. Pharmacol..

[14] Li C., Luo Y., Zhang W., Cai Q., Wu X., Tan Z., Chen R., Chen Z., Wang S., Zhang L. (2021). A comparative study on chemical compositions and biological activities of four essential oils: *Cymbopogon citratus* (DC.) Stapf, *Cinnamomum cassia* (L.) Presl, *Salvia japonica* Thunb. and *Rosa rugosa* Thunb. J. Ethnopharmacol..

[15] Pandey D.K., Chaudhary R., Dey A., Nandy S., Banik R., Malik T., Dwivedi P. (2020). Current knowledge of *Cinnamomum species*: A review on the bioactive components, pharmacological properties, analytical and biotechnological studies. Bioact. Nat. Prod. Drug Discov..

[16] Ma J., Chen X., Xue R., Wang F., Dong J., Tao N., Qin Z. (2023). Cinnamaldehyde inhibits cytokine storms induced by the ORF3a protein of SARS-CoV-2 via ROS-elimination in activated T cells. Phytother. Res..

[17] Shen C., Christensen L., Bak S., Christensen N., Kragh K. (2020). Immunomodulatory effects of thymol and cinnamaldehyde in chicken cell lines. J. Appl. Anim. Nutr..

[18] Harikrishnan R., Devi G., Balasundaram C., Van Doan H., Jaturasitha S., Saravanan K., Ringø E. (2021). Impact of cinnamaldehyde on innate immunity and immune gene expression in *Channa striatus* against *Aphanomyces invadans*. Fish Shellfish Immunol..

[19] El-Hamid M.I.A., El-Malt R.M., Al-Khalaifah H., Al-Nasser A., Elazab S.T., Basiony A., Ali A.M., Mohamed D.I., Nassan M.A., Ibrahim D. (2024). Exploring the interactive impacts of citronellol, thymol, and trans-cinnamaldehyde in broilers: Moving toward an improved performance, immunity, gastrointestinal integrity, and *Clostridium perfringens* resistance. J. Appl. Microbiol..

[20] Li Q., Ning N., Shen M., Xu H.-H., Wang L., Ding B.-Y., Hou Y.-Q., Guo S.-S. (2023). Effects of compound plant essential oil on growth performance and immune function of broilers challenged by *Clostridium perfringens*. Feed. Ind..

[21] Molenaar R.J., Dijkman R., Ter Veen C., Heuvelink A., van Kaam F., Augustijn M., Feberwee A. (2024). A Salmonella Pullorum outbreak with neurological signs in adult layers and outbreak investigation using whole genome sequencing. Avian Pathol..

[22] Saggar S., Mir P.A., Kumar N., Chawla A., Uppal J., Kaur A. (2022). Traditional and herbal medicines: Opportunities and challenges. Pharmacogn. Res..

[23] Momtaz S., Navabakhsh M., Bakouee N., Dehnamaki M., Rahimifard M., Baeeri M., Abdollahi A., Abdollahi M., Farzaei M.H., Abdolghaffari A.H. (2021). Cinnamaldehyde targets TLR-4 and inflammatory mediators in acetic-acid induced ulcerative colitis model. Biologia.

[24] Banerjee S., Banerjee S. (2023). Anticancer potential and molecular mechanisms of cinnamaldehyde and its congeners present in the cinnamon plant. Physiologia.

[25] Zhao H., Wu H., Duan M., Liu R., Zhu Q., Zhang K., Wang L. (2021). Cinnamaldehyde improves metabolic functions in streptozotocin-induced diabetic mice by regulating gut microbiota. Drug Des. Dev. Ther..

[26] El-Atawy M.A., Hanna D.H., Bashal A.H., Ahmed H.A., Alshammari E.M., Hamed E.A., Aljohani A.R., Omar A.Z. (2024). Synthesis, Characterization, Antioxidant, and Anticancer Activity against Colon Cancer Cells of Some Cinnamaldehyde-Based Chalcone Derivatives. Biomolecules.

[27] Wang M., Qazi I.H., Wang L., Zhou G., Han H. (2020). Salmonella virulence and immune escape. Microorganisms.

[28] Huang F.-C. (2021). The interleukins orchestrate mucosal immune responses to salmonella infection in the intestine. Cells.

[29] Kaminsky L.W., Al-Sadi R., Ma T.Y. (2021). IL-1β and the intestinal epithelial tight junction barrier. Front. Immunol..

[30] Kuo W.T., Odenwald M.A., Turner J.R., Zuo L. (2022). Tight junction proteins occludin and ZO-1 as regulators of epithelial proliferation and survival. Ann. N. Y. Acad. Sci..

[31] Beutel O., Maraspini R., Pombo-Garcia K., Martin-Lemaitre C., Honigmann A. (2019). Phase separation of zonula occludens proteins drives formation of tight junctions. Cell.

[32] Ma T.Y., Nighot P., Al-Sadi R. (2018). Tight junctions and the intestinal barrier. Physiology of the Gastrointestinal Tract.

[33] Thakur A., Mikkelsen H., Jungersen G. (2019). Intracellular pathogens: Host immunity and microbial persistence strategies. J. Immunol. Res..

[34] Van Liefferinge E., Forte C., Degroote J., Ovyn A., Van Noten N., Mangelinckx S., Michiels J. (2022). In vitro and in vivo antimicrobial activity of cinnamaldehyde and derivatives towards the intestinal bacteria of the weaned piglet. Ital. J. Anim. Sci..

[35] Kang R., Wang W., Liu Y., Huang S., Xu J., Zhao L., Zhang J., Ji C., Wang Z., Hu Y. (2022). Dietary selenium sources alleviate immune challenge induced by Salmonella Enteritidis potentially through improving the host immune response and gut microbiota in laying hens. Front. Immunol..

[36] Round J.L., Mazmanian S.K. (2009). The gut microbiota shapes intestinal immune responses during health and disease. Nat. Rev. Immunol..

[37] Yadav S., Jha R. (2019). Strategies to modulate the intestinal microbiota and their effects on nutrient utilization, performance, and health of poultry. J. Anim. Sci. Biotechnol..

[38] Ducatelle R., Goossens E., Eeckhaut V., Van Immerseel F. (2023). Poultry gut health and beyond. Anim. Nutr..

[39] Nagatake T., Kishino S., Urano E., Murakami H., Kitamura N., Konishi K., Ohno H., Tiwari P., Morimoto S., Node E. (2022). Intestinal microbe-dependent ω3 lipid metabolite αKetoA prevents inflammatory diseases in mice and cynomolgus macaques. Mucosal Immunol..

[40] Lu M., Xie L., Yin S., Zhou J., Yi L., Ye L. (2024). The Gut Microbial Lipid Metabolite 14 (15)-EpETE Inhibits Substance P Release by Targeting GCG/PKA Signaling to Relieve Cisplatin-Induced Nausea and Vomiting in Rats. J. Microbiol. Biotechnol..

[41] Li S., Duan X., Zhang Y., Zhao C., Yu M., Li X., Li X., Zhang J. (2024). Lipidomics reveals serum lipid metabolism disorders in CTD-induced liver injury. BMC Pharmacol. Toxicol..

[42] Li H., Pan C., Wang F., Li Z., Shahzad K., Huang Y., Zhao W. (2024). Multi-omics reveals the effects of dietary supplementation with Bupleuri radix branch powder on gut microbiota and lipid metabolism: Insights into gut microbial-muscle interactions. Microbiol. Spectr..

[43] Ma J., Mahfuz S., Wang J., Piao X. (2021). Effect of dietary supplementation with mixed organic acids on immune function, antioxidative characteristics, digestive enzymes activity, and intestinal health in broiler chickens. Front. Nutr..

[44] Dai D., Qiu K., Zhang H.-J., Wu S.-G., Han Y.-M., Wu Y.-Y., Qi G.-H., Wang J. (2021). Organic acids as alternatives for antibiotic growth promoters alter the intestinal structure and microbiota and improve the growth performance in broilers. Front. Microbiol..

[45] Abd El-Ghany W.A. (2024). Applications of Organic Acids in Poultry Production: An Updated and Comprehensive Review. Agriculture.

[46] Entrenas-García C., Argüello H., Calderón-Santiago M., Priego-Capote F., Zaldívar-López S., Garrido J.J. (2024). Salmonella typhimurium Alteration of Small Intestine Metabolism Early after Infection Is Linked to Microbiome Changes.

[47] Wang Y., Wu C., Gao J., Du X., Chen X., Zhang M. (2021). Host metabolic shift during systemic Salmonella infection revealed by comparative proteomics. Emerg. Microbes Infect..

[48] Hu D., Yang X., Qin M., Pan L.a., Fang H., Chen P., Ni Y. (2024). Dietary bile acids supplementation protects against Salmonella Typhimurium infection via improving intestinal mucosal barrier and gut microbiota composition in broilers. J. Anim. Sci. Biotechnol..

[49] Cai J., Chen X., Xu C., Zhu X., Wang H., Wu S., Cai D., Fan H. (2024). The Metabolic Pathway of Bile Secretion Is Vulnerable to Salmonella enterica Exposure in Porcine Intestinal Epithelial Cells. Animals.

[50] Zhang M., Wang Y., Zhao X., Liu C., Wang B., Zhou J. (2021). Mechanistic basis and preliminary practice of butyric acid and butyrate sodium to mitigate gut inflammatory diseases: A comprehensive review. Nutr. Res..

[51] Deters B.J., Saleem M. (2021). The role of glutamine in supporting gut health and neuropsychiatric factors. Food Sci. Hum. Wellness.

[52] Chen L., Zhong Y., Ouyang X., Wang C., Yin L., Huang J., Li Y., Wang Q., Xie J., Huang P. (2023). Effects of β-alanine on intestinal development and immune performance of weaned piglets. Anim. Nutr..

[53] Xu Q., Hu M., Li M., Hou J., Zhang X., Gao Y., Chachar B., Li X. (2021). Dietary bioactive peptide alanyl-glutamine attenuates dextran sodium sulfate-induced colitis by modulating gut microbiota. Oxidative Med. Cell. Longev..

[54] Liang H., Jiang F., Cheng R., Luo Y., Wang J., Luo Z., Li M., Shen X., He F. (2021). A high-fat diet and high-fat and high-cholesterol diet may affect glucose and lipid metabolism differentially through gut microbiota in mice. Exp. Anim..

[55] Villette R., Kc P., Beliard S., Salas Tapia M.F., Rainteau D., Guerin M., Lesnik P. (2020). Unraveling host-gut microbiota dialogue and its impact on cholesterol levels. Front. Pharmacol..

[56] Zhang F., Fan D., Huang J.-l., Zuo T. (2022). The gut microbiome: Linking dietary fiber to inflammatory diseases. Med. Microecol..

[57] Hays K.E., Pfaffinger J.M., Ryznar R. (2024). The interplay between gut microbiota, short-chain fatty acids, and implications for host health and disease. Gut Microbes.

[58] Liu L., Ling H., Zhang W., Zhou Y., Li Y., Peng N., Zhao S. (2022). Functional comparison of Clostridium butyricum and sodium butyrate supplementation on growth, intestinal health, and the anti-inflammatory response of broilers. Front. Microbiol..

[59] Ling Z.-N., Jiang Y.-F., Ru J.-N., Lu J.-H., Ding B., Wu J. (2023). Amino acid metabolism in health and disease. Signal Transduct. Target. Ther..

[60] Dai Z., Wu Z., Zhu W., Wu G. (2022). Amino acids in microbial metabolism and function. Recent Adv. Anim. Nutr. Metab..

[61] Peterson C.T., Perez Santiago J., Iablokov S.N., Chopra D., Rodionov D.A., Peterson S.N. (2022). Short-chain fatty acids modulate healthy gut microbiota composition and functional potential. Curr. Microbiol..

[62] Krautkramer K.A., Fan J., Bäckhed F. (2021). Gut microbial metabolites as multi-kingdom intermediates. Nat. Rev. Microbiol..

[63] Liu Y., Hou Y., Wang G., Zheng X., Hao H. (2020). Gut microbial metabolites of aromatic amino acids as signals in host–microbe interplay. Trends Endocrinol. Metab..

[64] Yoon J.-H., Do J.-S., Velankanni P., Lee C.-G., Kwon H.-K. (2023). Gut microbial metabolites on host immune responses in health and disease. Immune Netw..

