# Supplementary material for: Cinnamaldehyde Alleviates Salmonellosis in Chicks by Regulating Gut Health
Source: Vet Sci. 2025 Mar 3;12(3):237. doi: 10.3390/vetsci12030237 (PMC11946600; doi:10.3390/vetsci12030237)
Supplement: Supplementary file 1 [file vetsci-12-00237-s001.zip › vetsci-3418756-supplementary.pdf]

Supplementary Materials

In preliminary research, we have explored the therapeutic effects of different doses of Cinnamaldehyde(CA). Through survival rate experiments and clinical behavioral manifestations,it is pointed out that the treatment effect is best at a dose of 100mg/kg. In experiments, there were five experimental groups: control group, challenge group, and 50mg/kg CA,100mg/kg CA,and 150mg/kg CA treatment group. CA(50mg/kg, 100mg/kg,150mg/kg) was orally administered 6 hours after *Salmonella* infection, administer continuously for 3 days, once a day. Determine the dosage of medication by examining the survival rate of chicks infected with *Salmonella*.

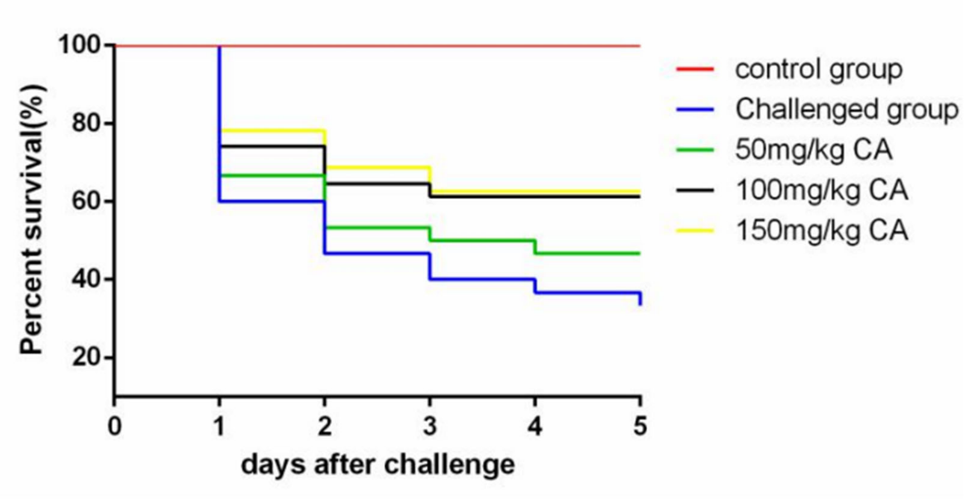

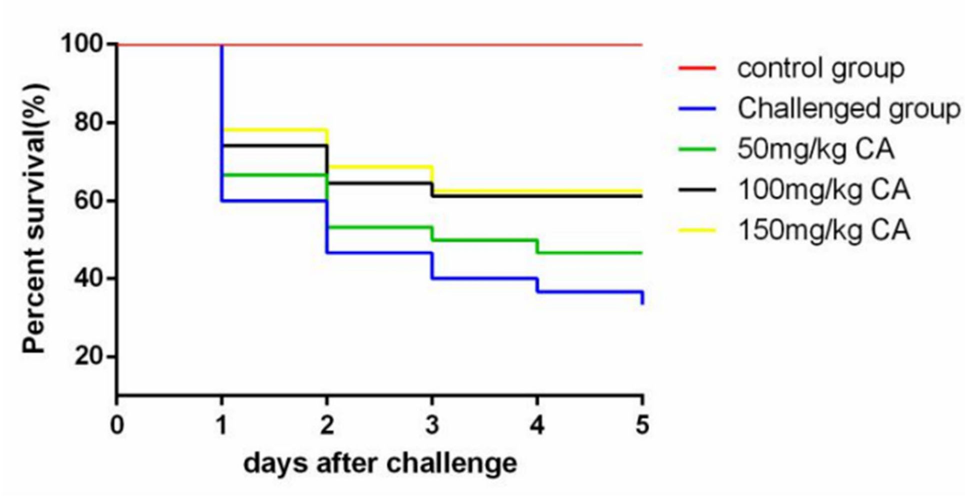

Fig S1 The daily survival rate of chicks from 1 to 5 days in different groups

The survival rate of chicks infected with Salmonella is 40%, while the survival rate after treatment with CA ranges from 50-80%. Among them, the 100mg/kg and 150mg/kg CA groups have the highest survival rates and the difference is not significant.

Observe and record the clinical behavior of different experimental groups of chicks, and

grade them according to the following table 1.Table 2 shows the clinical manifestations of chicks in different groups.

Table S1 Clinical behavior observation grading table

Grade	Behavioral Expression
1	breathing difficulties,acute death
2	diarrhea, discharge of white watery stool, and blockage of the anus
3	mental exhaustion, shrinking head and neck, hanging head and standing in a daze with closed eyes, sleeping soundly, with loose fur
4	loss of appetite, liking to pile up
5	Good mental state and active behavior

Table S2 Clinical behavior of chicks in each group

After Salmonella infection	Day1	Day2	Day3	Day4	Day5
control group	5	5	5	5	5
challenge group	1	2	3	3	3
50mg/kg CA	1	2	3	4	4
100mg/kg CA	1	3	4	5	5
150mg/kg CA	1	3	4	5	5

Chicks experience difficulty breathing 6 hours after being infected with Salmonella,in the following days, the chicks showed loss of appetite, a tendency to pile up, mental fatigue, shrinking of the head and neck, drooping of the head and standing still with closed eyes, drowsiness, messy villi, diarrhea, and white sticky feces at the hepatic hilum.The incidence of the treatment group was consistent with that of the challenge group. After oral administration, the condition improved on the 2nd and 3rd days, with slightly increased vitality and increased frequency of eating. The condition of diarrhea has improved and the frequency of bowel movements has decreased. On the 4th to 5th day, the situation significantly improved. After 5 days, the condition was basically eliminated. Taking into account the daily survival rate, clinical activity performance, and dosage of the chicks, the optimal dosage of cinnamaldehyde was determined to be 100mg/kg.
